# Leptospirosis Prevalence in a Population of Yucatan, Mexico

**DOI:** 10.4061/2011/408604

**Published:** 2011-11-02

**Authors:** Navarrete Espinosa Joel, Moreno Muñoz Maribel, Rivas Sánchez Beatriz, Velasco Castrejón Oscar

**Affiliations:** ^1^Coordinación de Vigilancia Epidemiológica y Apoyo en Contingencias, IMSS, México, DF, Mexico; ^2^Unidad de Medicina Experimental, Facultad de Medicina, UNAM, Mexico

## Abstract

*Objective*. To measure the prevalence of leptospirosis with two techniques in inhabitants of Izamal, Yucatan and to determine its relation with some exposure factors. *Material and Methods*. Transversal study in populations belonging to the HR62—IMSS-Opportunities working force in Izamal, Yucatan. Population, including 6 years of age or more, was randomly selected to participate in the study. A questionnaire was applied for personal ID and exposure factors; blood samples were taken for leptospirosis diagnosis. Simple frequencies, proportions, tendency and dispersion measures, prevalence and odd ratios and confidence intervals (CI) of 95%, and logistic regression model were obtained. *Results*. 204 patients, between 9 and 80 years old were included; 180 were positive (88.2%) with the dark-field technique; using MAT cutoff at 1 : 40, 178 patients (87.3%) were positive, while at 1 : 80 there were 103 positive (50.5%). The predominant serovar was Hardjo (94%). The highest prevalence was in women (96.3%) and in the >45-year-old group (95.7%); feminine gender (RM = 2.31 IC 95% 3.59–28.6), housewife (RM = 22.8 IC 95% 4.9–106.1), being in contact with stagnant water (RM = 5.2 IC 95% 1.7–15.9), and being in contact with domestic animal feces (RM = 5.1 IC 95% 1.9–13.1), these being the most significant variables in the final logistic regression model. *Conclusions*. The prevalence found was higher than the one nationally and internationally reported, representing an important finding, being in turn a local public health, maybe nationally. It is urgent to reinforce this research as well as to establish preventive and control measure to avoid exposure and health damages.

## 1. Introduction

Leptospirosis is a bacterial infection transmitted from animals to humans by direct contact through skin or mucous membranes with urine and other fluids from domestic or wild infected animals. The infection is usually present all year round, but it is more frequent during the rainy season, for the bacteria may survive for several weeks in humid, hot, and slightly alkaline environments. The clinical manifestation of the disease goes from an asymptomatic stage to a grave or even deadly state; currently two principal types of leptospirosis are differentiated: acute and chronic leptospirosis [1–3].

Risk populations are commonly described as adolescents, adults, country men, and the poorest urban populations, being also associated with raising animals in the home area, close relation with dogs and/or cats, handling excrement without protection, manipulation of beef or pork guts, lesions in feet during flooding, the use of sandals, and performing recreational activities allowing contact with stagnant water (swimming) [4–6]. 

The differential diagnosis includes a wide variety of infectious and noninfectious, acute and chronic diseases such as dengue, influenza, yellow fever, viral hepatitis, rheumatic, and oncologic diseases, and of the Central Nervous System [1–3].

In spite of being considered by the WHO as one of the most extended zoonose worldwide [7], it is suspected that there is an important underregistration. Except for the Antarctic, all continents regularly register cases, especially in tropical and subtropical regions [8].

During the last years, the recording in Mexico of this disease has increased as well as the number of states that report it [9]. The initial reports date from the first decade of the past century in Veracruz and Yucatan; currently, antibodies have been detected in half of the states of Mexico [4]. At the Instituto Mexicano del Seguro Social (IMSS) (Social Health Services), the reporting of cases started in 1999 and it is currently classified as a transcendental disease and its notification is mandatory [10, 11].

The reported infection prevalence is variable, due to, among other things, the use of different laboratory tests to estimate it and the use of different cutoff values for its interpretation [12]. This makes one consider some problems in its measurement, with an important underestimation.

Currently, it is known that leptospirosis shares an ecologic niche with other diseases and the presence of overlapped breakouts have been reported in areas where it overlaps with dengue. Due to the unspecific symptomatology of both infections, they are frequently mixed up, which results in a problematic diagnosis and treatment [1].

The state of Yucatan has all the ecological conditions to harbor this disease in its population; however, the morbidity reported in the region is low [9, 10]. The population of Izamal is located in Yucatan, a subrural community, descendent from the Mayas, and very close to Merida (capital state), with an average year-round temperature between 24° and 28°C. The average temperature in the coldest month is of 18°C, with a total annual precipitation of 700–1000 millimeters of rain. The objective of the present study was to estimate the prevalence of leptospirosis infection in this community.

## 2. Material and Methods

A prospective transversal study [13] was performed in inhabitants treated at the Rural Hospital number 62, at Izamal locality during 2007. The subjects, older than 6 years of age, were randomly selected, one out of ten subjects in the benefit list of the Opportunity Program from the IMSS. They were invited to participate voluntarily, for which informed specific information was granted and informed consent was required. 

The participants were visited in their homes and each answered a questionnaire to know their personal information and to explore some risk factors regarding the infection. Blood samples (7 mL vein blood) were taken with vacutainer, and total blood and serum were separated and refrigerated until they were sent to the laboratory for reference. For the diagnosis of the diseases, Microscopic Agglutination Tests (MAT) and Direct Observation of Dark Field [14, 15] were performed at the Laboratory of Tropical Medicine Department at the Experimental Medicine Unit in the Medicine Faculty at the Universidad Nacional Autónoma de México (UNAM).

To analyze the data, once the exploration analysis was performed, simple and proportional frequencies were calculated for all variables. For continual variables, normality was verified and then central tendency (mean) and dispersion measures (standard deviation) were applied. In the bivariate analysis, prevalence and odds ratio prevalence with confidence intervals (CI) at 95% were calculated. As association measures Mantel-Haenszel test was used and as effect measures, prevalence odds ratio with a CI at 95% were used.

To know the independent effect of each variable adjusted by the presence of other variables of interest, a logistic regression model [16] was constructed using the SPSS statistic package version 13, EPIINFO version 6, and Epidat version 3.1.

## 3. Results

From a total of 9,540 inhabitants, surveys and samples were obtained from 240 individuals. 

Sixty-eight individuals (33%) were males and one hundred and thirty-six (67%) were females. The average age was of 38.6 years old, with a mean of 38, ranging from 9 to 82 years. The age groups that reported a higher percentage in the samples were (a) the 25–44 years group (46.6%) and (b) older than 45 years group (34.2%).

Regarding schooling, 86 persons (42.2%) are illiterate, 59 (28.9%) did not finish elementary school, 30 (14.7%) with complete elementary, and 29 (14.2%) with other levels. 

Regarding household, 81.4% owned a house with 93.1% concrete or any other kind of material floor. Barely more than half the studied population (56.9%) had a complete bathroom; 27.5% defecated on the ground and 15.6% had a latrine. Most of them (94.1%) had piped water service and 5.9% used water that comes from wells or that is delivered by water tankers. From this population, 94.1% lived in areas where streets were not paved and 70% lived in overcrowded homes.

According to the socioeconomic level index, 82.5% had a low index, 16.6% had a medium index, and 0.9% had a high socioeconomic level index.

The highest proportion of subjects referred being housewives (61%), 48.5% of participants referred handling raw animal meat and guts, and 97% of these indicated they did not use protective gloves; in the same way, 48.5% performed agricultural labors, and from these, 95% were in contact with stagnant water, either barefoot or in contact with moist soil.

From this population, 88.2% had domestic animals living in the house, 78.9% reported direct contact with animal excrement; 73.5% referred having seen rodents in their house or their droppings in the yards.

Leptospirosis prevalence reported by the dark-field microscopic technique was of 88.2% (CI 95% 84.2–93.4), in general population. With the MAT technique and cut value of *⩾*1 : 80, the prevalence was of 50.5% (IC 95% 43.4–57.5); Table 1 shows the percentages of different cutoff values with this technique.

Predominant serovars were Hardjo with 94%, followed by Icterohaemorrhagiae with 3%, Pomona serovar had third place with 2%, and Canicola and Shermani serovars both with 0.5%. Regarding the number of serovars, 92% of the infected was positive to one serovar; meanwhile, 7% were positive to two; only 1% of the population was positive to three of more serovars.

The prevalence was 96.3% (IC 95% 91.6–98.8) in women and 72.1% (CI 95% 60.7–83.5) in men.

The most affected age group was the one 45 or more years old with 95.7% (CI 95% 87.9–99.1), followed by the 25–44 year of age with 87.3%. The group 5–14 years old presented 80% (CI 95% 28.3–99.5) and the 15–24 years old presented 76% (CI 95% 60.7–83.5).

According to schooling, the group with incomplete elementary school had 93.2% (IC 95% 83.5–98.1) affectation, followed by the group with other studies 93.1% (CI 95% 77.2–99.2), and the group with complete elementary school with 86.7% (CI 95% 69.3–96.2); illiterates showed a prevalence of 83.7% (CI 95% 74.2–91.2). 

Regarding housing features, 92% (CI 95%, 82.7–98.0) of those who defecated on open ground, resulted positive to leptospirosis. In the same way, 90% (CI 95% 74.9–98.0) of those having latrines, and only 85% (CI 95% 78.5–92.2) of those having complete bathrooms.

In relation to their occupation, housewives were the most affected with 98% (CI 95% 94.3–99.8) of infected; meanwhile, 87.7% (CI 95% 80.9–94.8) of those in contact with animal guts were positive. The highest prevalence among those who referred performing agricultural activities 89.9% (CI 95% 82.1–95.9) and those in contact with stagnant water 89.5% (CI 95% 82.3–96.1) (Table 2).

From the participants having domestic animals in their homes, 87.2% (CI 95% 82.0–92.3) were positive to the disease, and from those who were in contact with the animals' excrements, 92.3% (CI 95% 87.5–97.0) were positive. Of the persons who reported seeing rodents in their houses, 90% (CI 95% 84.8–95.1) were positive to leptospirosis (Table 3).

In the bivariate analysis, women had 10 times more risk of infection with leptospirosis when compared with men (RMP = 10.1, CI 95% 3.6–28.6, *P* < 0.05).

Although there was no significant statistical association, the 45-year-old group was almost 6 times (RMP = 5.58, CI 95% 0.4–66, *P* = 0.1) more at risk of leptospirosis infection when compared with the group of 5–14 years old, and the 25–44-year-old group had a 70% (RMP = 1.7, CI 95% 0.2–16.7, *P* = 0.1) more risk of infection when compared to the same group.

Also, in regards to handling excrement, those who defecated at open sky, had two times more risk to get infected (RMP = 2.23, CI 95% 0.7–6.9, *P* > 0.05) when compared with those who had bathroom. Meanwhile, those with latrine presented 60% (RMP = 1.6, CI 95% 0.4–6.0, *P* > 0.5) more probabilities of getting sick when compared with those having complete bathrooms. 

The individuals reporting having performed labors in the fields had 30% more risk of infection when compared with those who did not labor in the fields (RMP = 1.3, CI 95% 0.5–2.6, *P* > 0.05). From these, those in contact with stagnant water, either by walking in it barefoot or manipulating humid soil, had two time more risk of getting sick than the group referring no contact (RMP = 2.8, CI 95% 0.2–29.8, *P* > 0.05). Those reporting having contact with rodents and their droppings had an excess risk of getting sick of 80% when compared with the group with no rodents in their homes (RMP = 1.8, CI 95% 0.7–4.3, *P* > 0.05).

Otherwise, from those who referred having contact with domestic animal excrement, 40% (RMP = 1.4, CI 95% 1.1–1.6, *P* < 0.05) had a higher probability of getting infected with leptospirosis when compared with the group who had no contact. 

For the multivariate analysis, the logistic regression model was applied, in which the most significant variables of the bivariate analysis were included (*P* < 0.1). The variables which better explained the presence of infection by Leptospira were being female and housewife, as well as being in contact with stagnant water and with excrement from domestic animals (Table 4).

Global concordance between the two used diagnostic techniques used in this study was highest with the cut off *⩾*1 : 40 in MAT (78%) (Table 5).

## 4. Discussion

Since the ending of the past century, it was foreseen that global warming in our planet would bring as a consequence changes in the ecology of many regions, and in turn, the alteration in presentation patterns of diseases, as well as the arousal and resurge of a large number of infectious diseases [17–19].

These facts are proven with the increase of some diseases as dengue, the resurge of cholera, and the surging of some diseases, such as leptospirosis, that, even though it is not recent, it is only a few years ago when it came under the spot light due to the breakouts of this diseases as consequence of weather phenomena, characterized by important floods, and which has been confused with other pathologies, such as dengue [7, 20, 21]. Facts are even more evident when we found the concurrence of diverse agents that produce overlapped outbreaks of different diseases as in the case of dengue and leptospirosis. This has very important repercussions regarding timely diagnosis and an effective treatment to patients. 

Leptospirosis is an infection with a magnitude that can be much more to the one known up to date. The disease may be present as acute or chronic; the first has a variable behavior and may cause death due to its graveness. These cases are generally part of the epidemiology numbers. However, the chronic cases of this disease are not generally recognized, much less notified nor quantified. 

Up to not long ago, it was recognized that the infection could behave in a chronic manner; nonetheless, nationwide findings and the results of the present study confirm this fact. Prevalence found in previous studies are less to what we report in this work, which could be due to differences in the studied population or the cutoff values established to consider infection; nevertheless, the direct observation performed in dark field demonstrated the presence of the bacteria in 8 of every 10 samples, which represents a high infection rate in the general population.

In this sense, the comparison of the microscopic agglutination test at different cutoff values against a direct observation in the dark field (gold standard test) has allowed us to identify the ideal cutoff values that make possible considering an individual as infected either in an acute or chronicle status. This aspect is of main importance for the cutoff value established by the Norma Oficial Mexicana (Mexican Official Norm) (1 : 80) that is useful for the identification of recent infections; however, it is inadequate to identify cases that are infected chronically, and that may propitiate an underestimation of the real infection prevalence in the general population.

These facts place leptospirosis as a local public health problem that might be of great importance nationwide, if we consider the large variety of pathologic processes in which leptospira may intervene to cause the disease [22, 23] and that in most of the country's entities there exist the convenient conditions for the infection to flourish, thus theimportance to establish active epidemiologic alert for this diseases in order to establish the real importance of this problem; it is probable we are just observing the tip of the iceberg. 

The prevalence that we found is higher than the one reported in other studies performed in Yucatan [24, 25] and the differences may be explained due to the techniques and cutoff values that were used; in this sense, the microscopic agglutination test is targeted to the detection of specific antibodies and demonstrates the contact of the individual with *leptospira *in a determined moment, especially in recent infection; however, it only detects the targeted serovar. In this way, the prevalence may be underestimated when the adequate antigens are not used. On the contrary, the direct observation in dark field identifies *Leptospira* without being precise to the observed serovar; therefore, both tests are complementary. Nevertheless, to identify an acute or chronic infection, the observation in dark field and culture [26] are ideal tests, and microscopic agglutination with a cutoff value of 1 : 40 may be an alternative. 

For all the above, the fact of not having found symptomatic individuals in the studied population, as well as the use of low antibody titers, leads us to conclude that a large proportion of the surveyed presented chronic asymptomatic infection which was determined by observation of the bacteria in dark field.

The risk factors that we found are consistent with other reports and are in close relation with labor conditions, insalubrious environment, and poverty, making a priority the health education to avoid contact with contaminated elements, as well as the need to impulse social infrastructure development with this same purpose in mind.

The presence of animals inside the households of the interviewed and the predominant serovars that were found show the direct contact among hogs, bovines, rats, and dogs and persons, a possible source of infection. Again, due to the above, information for self-protection is an important factor to avoid direct contact with animals and their droppings.

The constant report of simultaneous infections, confirmed to dengue and leptospira, in patients who were initially diagnosed with dengue, allows us to infer that the exposure and infection by both agents may be simultaneous, or, even more, that in a patient with a chronic infection by *Leptospira* and who suffers infection by dengue, *Leptospira *might act as an opportunistic agent and unleash an acute infection that worsens the prognostic of the patient, especially if there is no suspicion of leptospirosis, and offering antibiotic treatment on time may save his/her life. This is especially important if we consider that the rate with the highest success to eliminate an infection is offering antibiotics during the first days of the onset of the infection, which in turn might avoid the evolution of the infection to a chronic state. 

Finally, the results here presented may serve as the bases for the doctor when establishing at first contact the treatment for patients who are suspected of dengue with renal or liver complications, and in who may also be suspected of infection by leptospira or both, especially ifs the patient lives in an endemic area.

## Figures and Tables

**Table 1:** Leptospirosis prevalence by means of 2 diagnostic techniques.

Used technique	*n* (204)	%	CI 95%
Dark Field	180	88.2	83.6–92.1
MAT ≥ 1 : 40	178	87.3	82.4–92.1
MAT ≥ 1 : 80	103	50.5	43.4–57.5
MAT ≥ 1 : 160	27	13.2	6.3–18.1

**Table 2:** Leptospirosis prevalence and occupational exposure.

Variable	Positive	%	CI 95%	Negative	%	CI 95%
Occupation						
Housewife	123	98	94.3–99.8	2	1.6	0.19–5.7
Farmer	22	71	53.4–88.6	9	29	11.4–46.6
Other	35	92.1	78.6–98.3	3	7.9	1.7–21.4

Contact with raw meat						
Yes	87	87.7	80.9–94.8	12	12.3	5.1–19.0
No	93	88.6	82.0–95.1	12	11.4	4.8–17.9

Agricultural labor						
Yes	88	89.9	82.1–95.9	11	11.1	4.4–17.8
No	92	87.7	80.8–94.3	13	12.3	5.6–19.1

Contac with stagnant water						
Yes	85	89.5	82.3–96.1	10	10.5	3.8–17.2
No	3	75.0	.631–8.6	1	25.0	19.4–99.3

**Table 3:** Leptospirosis prevalence and contact with domestic animals.

Variable	Positive	%	CI 95%	Negative	%	CI 95%
Presence of animals in the household						
Yes	157	87.2	82.0–92.3	23	12.8	7.6–17.9
No	23	95.8	78.8–99.8	1	4.2	.10–21.1

Contact with domestic animals' excrement						
Yes	131	92.3	87.5–97.0	11	7.7	2–9–12.5
No	23	68.4	52.3–84.5	12	31.5	15.4–47.6

Contact with rodents and their droppings						
Yes	135	90	84.8–95.1	15	10.0	4.8–15.1
No	45	83.3	72.4–99.1	9	16.7	5.8–27.5

**Table 4:** Logistic regression model.

Variable	RMP	CI 95%	*P*
Gender			
Male	1		
Female	23.6	6.9–81.1	<0.05

Occupacion			
Other	1		
Housewife	22.8	4.9–106.1	<0.05

Contact with Stagnant Water			
No	1		
Yes	5.2	1.7–15.9	<0.05

Contact with Urine or Excrement			
No	1		
Yes	5.1	1.9–13.1	<0.05

**Table 5:** Concordance percentage between the 2 diagnostic techniques.

MAT cutoff values	Concordance percentage	Kappa percentage
>1 : 40	78	58
>1 : 80	50	19
>1 : 160	21	3
